# Supplementary material for: Postoperative Impact of Pontocerebellar Angle Surgery on the Quality of Life in Patients with Vestibular Schwannoma
Source: Audiol Res. 2022 Nov 17;12(6):635–43. doi: 10.3390/audiolres12060061 (PMC9680513; doi:10.3390/audiolres12060061)
Supplement: Supplementary file 1 [file audiolres-12-00061-s001.zip › audiolres-1991344-supplementary.pdf]

SUPPLEMENTARY FILE

STATISTICAL ANALYSIS, SUPPLEMENTARY TABLES AND DATA

Table S1: Subgroups based on postoperative facial dysfunction measured by HB score. Wilcoxon test was used (p (α) < 0,05)

	Normal or mild HB I - II (n = 19)		Severe HB IIII or > (n = 12)		W
	Median	SD	Median	SD	p (α)
Anxiety	62.5	26.44	59.375	26.69	n.s.
Facial Dysfunction	75	24.64	51	24.52	0.03*
General health	25	31.43	43.75	30.93	n.s.
Balance	50	33.14	50	33.73	n.s.
Hearing	37	20.79	40.62	20.58	n.s.
Energy	58.3	26.68	66.7	26.77	n.s.
Pain	75	38.14	47.5	38.04	0.014*
Total	52.9	20.4	54.95	20.67	n.s.

*Statistically significant after Bonferroni correction.
HB: House–Brackmann; n.s.: not significant; SD: standard deviation; W: Wilkoxon log rank test

Table S2: Subgroups based on preoperative tumor size measured by Koos classification. Wilcoxon test was used (p (α) < 0.05)

	Koos LOW GRADE (I -II)		Koos HIGH GRADE (III-IV)		W
	Median	SD	Median	SD	p (α)
Anxiety	62.5	31.25	59.37	25.10	n.s.
Facial Dysfunction	75	29.50	58.3	22.74	n.s.
General health	50	24.56	25	31.79	n.s.
Balance	33.33	39.00	52.08	30.28	n.s.
Hearing	31.25	21.52	40.62	20.23	n.s.
Energy	66.7	30.82	62.5	25.90	n.s.
Pain	75	37.73	75	37.74	n.s.
Total	59	26.75	52.9	18.02	n.s.

*Statistically significant after Bonferroni correction.
n.s., not significant; SD, standard deviation, W: Wilkoxon log rank test

Table S3: Subgroups based on postoperative complications. Wilcoxon test was used (p (α) < 0.05)

	No postop complications		Postop complications		KW
	Median	SD	Median	SD	p (α)
Anxiety	75	28.26	56.25	20.35	n.s.
Facial Dysfunction	70.85	27.25	58.3	17.20	n.s.
General health	50	30.77	25	28.64	n.s.
Balance	54.17	34.62	33.33	28.17	n.s.
Hearing	43.75	20.77	25	15.15	n.s.
Energy	66.7	27.70	50	24.72	n.s.
Pain	75	37.74	75	37.73	n.s.
Total	59.10	21.32	42.3	17.07	n.s.

W: Wilkoxon Log-Rank test
*Statistically significant after Bonferroni correction.
n.s., not significant; SD, standard deviation.

PREOPERATIVE HEARING LEVEL

Patients were assigned into four groups according to their preoperative hearing level on the hear where tumor was located, according to the classification system of the Committee on Hearing and Equilibrium of the American Academy of Otolaryngology-Head and Neck surgery (AAO-HNS) 1995: group A (n = 4) *PTA (Pure Tone Thresholds) ≤ 30 dB and SDS (Speech discrimination %) ≥ 70%;* group B (n = 5) *PTA > 30 dB, ≤ 50 dB and SDS ≥ 50%,* group C (n = 8) *PTA > 50 dB and SDS ≥ 50%,* group D (n = 14) *PTA any level and SDS < 50%.* There was not a specific trend for the domain of "hearing" into different groups, and even Kruskal-Wallis test showed no significative differences for every group.

	AUDITORY GROUP KRUSKALL-WALLIS TEST
PANQOL TOT	0.754 ns
BALANCE	0.231 ns
HEARING	0.731 ns
ANXIETY	0.512 ns
FACIAL DYS	0.771 ns
ENERGY	0.780 ns
PAIN	0.754 ns
GENERAL HEALTH	0.350 ns
